# Airborne metals and polycyclic aromatic hydrocarbons in relation to mammographic breast density

**DOI:** 10.1186/s13058-019-1110-7

**Published:** 2019-02-13

**Authors:** Alexandra J. White, Clarice R. Weinberg, Ellen S. O’Meara, Dale P. Sandler, Brian L. Sprague

**Affiliations:** 1 Epidemiology Branch, National Institute of Environmental Health Sciences, NIH, Research Triangle Park, NC 27709-2233 USA; 2 Biostatistics and Computational Biology Branch, National Institute of Environmental Health Sciences, NIH, Research Triangle Park, NC USA; 3 Kaiser Permanente Washington Health Research Institute, Seattle, WA USA; 4 Departments of Surgery and Radiology, University of Vermont, Burlington, VT USA

**Keywords:** Air pollution, Metals, Polycyclic aromatic hydrocarbons, Breast density, Breast cancer, BCSC

## Abstract

**Background:**

Breast density is strongly related to breast cancer. Identifying associations between environmental exposures and density may elucidate relationships with breast cancer. Metals and polycyclic aromatic hydrocarbons (PAHs) may influence breast density via oxidative stress or endocrine disruption.

**Methods:**

Study participants (*n* = 222,581) underwent a screening mammogram in 2011 at a radiology facility in the Breast Cancer Surveillance Consortium. Zip code residential levels of airborne PAHs and metals (arsenic, cadmium, chromium, cobalt, lead, manganese, mercury, nickel, and selenium) were assessed using the 2011 EPA National Air Toxics Assessment. Breast density was measured using the Breast Imaging–Reporting and Data System (BI-RADS) lexicon. Logistic regression was used to estimate adjusted odds ratios (ORs) and 95% confidence intervals (CI) for the individual air toxics and dense breasts (BI-RADS 3 or 4). Weighted quantile sum (WQS) regression was used to model the association between the air toxic mixture and density.

**Results:**

Higher residential levels of arsenic, cobalt, lead, manganese, nickel, or PAHs were individually associated with breast density. Comparing the highest to the lowest quartile, higher odds of having dense breasts were observed for cobalt (OR = 1.60, 95% CI 1.56–1.64) and lead (OR = 1.56, 95% CI 1.52–1.64). Associations were stronger for premenopausal women. The WQS index was associated with density overall (OR = 1.22, 95% CI 1.20–1.24); the most heavily weighted air toxics were lead and cobalt.

**Conclusions:**

In this first study to evaluate the association between air toxics and breast density, women living in areas with higher concentrations of lead and cobalt were more likely to have dense breasts.

**Electronic supplementary material:**

The online version of this article (10.1186/s13058-019-1110-7) contains supplementary material, which is available to authorized users.

## Background

Breast density, a marker of heightened breast cancer risk, may be influenced by environmental insults [1]. Women in the highest category of density tend to have a four to fivefold higher risk of breast cancer [2]. Elucidating the role the environment plays in breast cancer is an important area of research, as the incidence of breast cancer remains high [3] and most identified risk factors for breast cancer are not modifiable [4]. Evaluating environmental predictors of breast density may provide more proximal evidence to support a role of chemicals in carcinogenesis and also suggest potential biologic mechanisms of importance.

Although genetics is an important determinant of breast density [5], accumulating evidence suggests that lifestyle and environmental factors, such as hormone therapy (HT) use and cigarette smoking [2, 6–9], may also influence breast density. Previous studies have considered the relationship between air pollution and other environmental chemicals and breast density with inconsistent results [1, 10–14]. Breast density declines after discontinuing HT or with tamoxifen use [15–17], underscoring the modifiability of this risk factor and the potential value of identifying environmental determinants of density.

Both polycyclic aromatic hydrocarbons (PAHs) and heavy metals are environmental endocrine disruptors and can induce oxidative stress that may influence the risk of breast cancer. Sources of PAH exposure [18], including exposure to traffic pollution [19], have been previously related to breast cancer risk, and studies of air toxics have also suggested a role for airborne metals in breast cancer [20, 21]. PAHs are ubiquitous environmental contaminants formed by the combustion of organic material. PAHs have been shown to exhibit both estrogenic and anti-estrogenic effects [22]. In addition, evidence from experimental studies supports the hypothesis that many toxic metals may act as endocrine disruptors, and as such, they are often referred to as “metalloestrogens” [23]. Given these biologic mechanisms, it is plausible that these exposures may also impact breast density.

The aim of this study was to evaluate the relationship between living in areas of higher airborne metals and PAHs and breast density, with exposures assessed both individually, and using a mixture approach. We hypothesized that women residing in an environment with higher levels of air toxics would be more likely to have dense breasts.

## Methods

### Study population

This study utilized the National Cancer Institute’s Breast Cancer Surveillance Consortium (BCSC) [24]. The BCSC is a collaborative network of mammography registries (www.bcsc-research.org). Five registries provided data for the study: Carolina Mammography Registry, Vermont Breast Cancer Surveillance System, Kaiser Permanente Washington Registry (Washington State), San Francisco Mammography Registry, and New Hampshire Mammography Network. This resource was designed to assess breast cancer screening and patient outcomes from a geographically diverse sample of over 100 community radiology facilities. Women undergoing mammograms at these facilities complete a health questionnaire at each breast imaging exam, which includes items on sociodemographic characteristics, medical history, and breast cancer risk factors. Distributions of sociodemographic characteristics and race in women in BCSC counties are similar to those of the US population [24]. Each registry and the Statistical Coordinating Center (SCC) received institutional review board approval for either passive or active consenting processes or a waiver of consent to enroll participants, link data, and perform analytic studies. All procedures are Health Insurance Portability and Accountability Act (HIPAA) compliant, and all registries and the SCC have received a federal Certificate of Confidentiality and other protection for the identities of women, physicians, and facilities who are subjects of this research.

Since 1994, the BCSC has prospectively collected data on mammograms conducted at participating radiology facilities. Breast density information was recorded in clinical practice by the interpreting radiologist using the standard clinical scoring information as determined by the interpreting radiologist using Breast Imaging–Reporting and Data System (BI-RADS) categories (1, entirely fatty; 2, scattered areas of fibroglandular density; 3, heterogeneously dense; 4, extremely dense). Available data elements include demographic characteristics (zip code, age, race, and education), reproductive characteristics (parity), health history (family and personal history of breast cancer), screening mammography history, use of HT, and menopausal status. Zip code level data on income, poverty, and education was derived from the US Census.

The current analysis was limited to women with no personal history of breast cancer who underwent a routine screening mammogram in 2011 and who were not missing either breast density or residential zip code information (*n* = 285,817 women). If a woman had more than one mammogram in 2011, we selected the first mammogram of that calendar year.

### Exposure assessment

Levels of airborne toxics were assessed using the Environmental Protection Agency (EPA) National Air Toxics Assessment (NATA), a database that provides information on concentrations of toxic air pollutants nationally. The 2011 NATA is the most recently released version of the data. NATA assessed levels of air toxics by using validated air pollution models that utilizes input from the National Emissions Inventory, a comprehensive compilation of information on major stationary sources (factories, incinerators), area and other sources (dry cleaners, small manufacturers), on-road and non-road mobile sources (cars, trucks and boats), events (wildfires), and biogenics (naturally occurring emissions). The validated air pollution model also incorporates secondary information, specifically the formation of secondary pollutants from reactions between pollutants, and background exposure levels from long-range transport from distant sources [25].

NATA provides census-tract level estimates for polycyclic organic matter, which is predominately composed of PAH [26] in addition to PAH derivatives [27], and for the metals arsenic, cadmium, chromium, cobalt, lead, manganese, mercury, nickel, and selenium. BCSC collects information on residential zip code at the time of the mammogram. To link the BCSC data with the NATA exposure information, we used the US Department of Housing and Urban Development zip code crosswalk files to link the census track airborne toxic estimates to the residential zip code [28].

### Statistical analysis

The primary analysis was a cross-sectional study to estimate the association between living in a zip code with higher exposure to individual airborne toxics and breast density in 2011. Air toxic exposure levels were categorized based on quartiles, and the same cut points were used consistently throughout all analyses except for a sensitivity analysis for select air toxics in which they were characterized using deciles. Breast density was classified using the BI-RADS categories. Descriptive characteristics and Pearson correlation coefficients (*r*) were estimated. Unconditional logistic regression was used to estimate odds ratios (ORs) and 95% confidence intervals (CI) when BI-RADS categories were collapsed to a dichotomous variable with BI-RADS 1 or 2 classified as non-dense and BIRADS 3 or 4 as dense. Multinomial regression was used when the outcome was the four-level BI-RADS categories.

We evaluated effect measure modification of the association between air toxics and breast density by menopausal status and hormone therapy use by including a cross-product term in the model and testing the statistical significance using a likelihood ratio test. We also considered whether PAH or selenium exposure modified the association between the remaining airborne metals and density. These specific exposures were selected as potential effect measure modifiers because selenium has been hypothesized to counteract the negative impacts of metals such as cadmium [29] and PAHs have been hypothesized to act synergistically with toxic metals via induction of oxidative stress [30]. The confounder adjustment set was based on consideration of directed acyclic graphs [31] and included age (≤ 45, 46–50, 51–55, 61–65, 66–69, ≥ 70), race/ethnicity (white, black, Asian/native Hawaiian/Pacific Islander, Hispanic, other), parity (ever/never), and zip code level income (< $50,000, $50,000–$99,999, ≥ $100,000), and education. We did a complete case analysis, limiting to women who did not have missing values for the variables in the adjustment set (*n* = 222,581 women).

In sensitivity analyses, we considered further covariate adjustment for rural/urban status, hormone replacement therapy, and BMI. BMI data were not collected at all study sites, and BMI was missing for many women (45%). Therefore, we examined the impact of adjusting for BMI as a confounder in analyses limited to BCSC participants with non-missing BMI. We additionally tested whether BMI was correlated with air toxic levels using Pearson correlation statistics.

As a secondary analysis, we used weighted quantile sum (WQS) regression to evaluate the relationship between the air toxic mixture and breast density overall. WQS has been described previously [32–34]. In this application, WQS was used to estimate a weighted linear index to evaluate the combined association of correlated air toxics classified in quartiles in relation to breast density*.* The data were randomly split into a training (40%) and validation (60%) dataset. The weights were empirically determined in the training dataset via bootstrap sampling (*n* = 100). In WQS, weights can range between 0 and 1 but are constrained to sum to 1 across the individual components of the mixture. If all air toxics received equal weights, the weight for each would be 0.1. Weights greater than 0.1 signify a higher contribution to the weighted index than expected; higher weights indicate stronger associations with breast density. The strengths of WQS include the estimation of an overall mixture effect as well as the identification of the exposures that appear to drive the association. WQS was selected as prior simulation studies have shown it to have a good sensitivity and specificity compared to other mixtures approaches [32, 33].

## Results

Of the 222,581 women who met our inclusion criteria, approximately 45% (*n* = 100,107) had dense breasts, defined as BI-RADS 3 or 4 (Table 1). Women were more likely to fall into the middle BI-RADS categories (2 and 3) than the extremes (1 and 4). As expected, women who were older or postmenopausal, and women with a higher BMI were more likely to have non-dense breasts. Asian women and women who were living in a zip code with a higher median income and education were more likely to have dense breasts.

**Table 1:** Study participant characteristics by breast density (Breast Cancer Surveillance Consortium, 2011)

	Non-dense (*N* = 122,474; 55%)		Dense (*N* = 100,107; 45%)	
	*N*	Row %	*N*	Row %
Age				
≤ 45	11,198	35	21,145	65
46–50	11,716	40	17,752	60
51–55	16,034	51	15,371	49
56–60	22,305	59	15,606	41
61–65	21,832	64	12,540	36
66–69	15,864	67	7794	33
≥ 70	23,525	70	9899	30
Race				
White	87,482	57	67,257	43
Black	13,720	62	8365	38
Asian, native Hawaiian or Pacific Islander	9846	37	16,612	63
Other	2847	56	2256	44
Hispanic	8579	60	5617	40
Body mass index				
< 18.5	535	21	2042	79
18.5–24.9 kg/m^2^	23,987	38	39,821	62
25–29.9 kg/m^2^	24,678	62	15,195	38
≥ 30 kg/m^2^	27,607	79	7383	21
Missing	45,667	56	35,666	44
Ever given birth				
No	23,636	48	25,165	52
Yes	98,838	57	74,942	43
Hormone therapy use				
No	106,627	55	88,228	45
Yes	5873	51	5614	49
Missing	9974	61	6265	39
Menopausal status				
Premenopausal	26,167	38	42,779	62
Postmenopausal	96,307	63	57,328	37
Zip code level median income				
< $50,000	40,119	62	24,347	38
$50,000–$99,999	74,132	53	65,067	47
≥ $100,000	8223	43	10,693	57
Zip code level college education				
Quartile 1	27,836	61	17,815	39
Quartile 2	33,424	58	24,535	42
Quartile 3	27,621	54	23,729	46
Quartile 4	33,593	50	34,028	50
BI-RADS				
1	28,617	100	0	0
2	93,857	100	0	0
3	0	0	80,462	100
4	0	0	19,645	100

Quartiles and ranges of exposure to air toxics are shown in Table 2. Median exposure levels were highest for PAH and mercury compared to the other air toxics. The air toxics were moderately correlated (Additional file 1: Table S1, with correlations ranging from − 0.01 to 0.86).

**Table 2:** Air toxic exposure distributions (μg/m^3^) (Breast Cancer Surveillance Consortium, 2011)

Air toxic	Minimum	25th percentile	50th percentile	75th percentile	Maximum
Arsenic	3.58E−06	3.08E−05	6.34E−05	1.20E−04	1.53E−03
Cadmium	5.16E−07	1.35E−05	3.02E−05	5.70E−05	2.43E−03
Chromium	6.32E−07	2.07E−05	6.60E−05	1.33E−04	3.49E−03
Cobalt	4.10E−05	4.30E−05	4.85E−05	5.59E−05	1.34E−03
Lead	2.49E−05	3.47E−04	6.42E−04	1.29E−03	4.02E−02
Manganese	1.91E−05	3.32E−04	5.56E−04	1.10E−03	6.17E−02
Mercury	1.03E−04	1.29E−03	1.49E−03	1.89E−03	1.58E−02
Nickel	9.60E−06	2.57E−04	5.27E−04	8.73E−04	3.71E−02
Selenium	2.00E−04	2.15E−04	2.87E−04	4.46E−04	3.74E−03
PAHs	1.53E−04	1.69E−03	3.15E−03	7.23E−03	6.66E−02

In general, living in areas with higher exposure to some, but not all, individual air toxics was associated with higher odds of having dense breasts (Table 3). Associations were most evident for women who lived in areas with high exposure to cobalt (quartile 4 (Q4) vs. quartile 1(Q1), OR = 1.60, 95% CI 1.56–1.64) and lead (Q4 vs Q1, OR = 1.56, 95% CI 1.52–1.60). Higher odds of dense breasts were also observed for women living in areas with higher exposure to arsenic (Q4 vs Q1, OR = 1.20, 95% CI 1.17–1.23), manganese (Q4 vs Q1, OR = 1.18, 95% CI 1.15–1.21), nickel (Q4 vs Q1, OR = 1.23, 95% CI 1.20–1.26), and PAH (Q4 vs Q1, OR = 1.27, 95% CI 1.23–1.31). Although we observed elevated ORs for the second and third quartiles of chromium and mercury exposure, the associations were not apparent in the fourth quartile. Little to no association was observed for higher quartiles of cadmium or selenium when compared to the lowest quartile. In sensitivity analyses using deciles to evaluate the dose–response trends for cobalt and lead, we found that the observed trends were relatively linear with increasing lead exposure (Additional file 1: Fig. S1). For cobalt, there was an initial inverse trend that then reversed and became positive after the median exposure level.

**Table 3:** Quartiles of air toxics and breast density (Breast Cancer Surveillance Consortium, 2011)

Air toxics	Non-dense breasts	Dense breasts	Age-adjusted OR (95% CI)	Adjusted OR (95% CI)^1^
Arsenic				
Quartile 1	31,220	26,086	1.00 (reference)	1.00 (reference)
Quartile 2	32,046	20,194	0.76 (0.74, 0.78)	0.79 (0.77, 0.81)
Quartile 3	27,568	26,764	1.16 (1.13, 1.19)	1.25 (1.21, 1.28)
Quartile 4	31,640	27,063	1.05 (1.02, 1.07)	1.20 (1.17, 1.23)
Cadmium				
Quartile 1	33,843	24,279	1.00 (reference)	1.00 (reference)
Quartile 2	28,330	22,972	1.07 (1.05, 1.10)	0.94 (0.92, 0.97)
Quartile 3	27,637	25,674	1.23 (1.20, 1.26)	1.00 (0.98, 1.03)
Quartile 4	32,664	27,182	1.11 (1.08, 1.14)	0.92 (0.90, 0.95)
Chromium				
Quartile 1	37,475	26,042	1.00 (reference)	1.00 (reference)
Quartile 2	28,046	21,201	1.07 (1.04, 1.10)	1.05 (1.02, 1.08)
Quartile 3	28,691	28,069	1.38 (1.35, 1.42)	1.21 (1.18, 1.24)
Quartile 4	28,262	24,795	1.21 (1.18, 1.24)	0.96 (0.93, 0.99)
Cobalt				
Quartile 1	36,555	20,137	1.00 (reference)	1.00 (reference)
Quartile 2	26,648	22,128	1.43 (1.39, 1.46)	1.18 (1.14, 1.21)
Quartile 3	28,415	27,756	1.73 (1.69, 1.77)	1.44 (1.40, 1.48)
Quartile 4	30,856	30,086	1.76 (1.72, 1.80)	1.60 (1.56, 1.64)
Lead				
Quartile 1	35,577	22,375	1.00 (reference)	1.00 (reference)
Quartile 2	29,904	24,019	1.27 (1.24, 1.30)	1.05 (1.03, 1.08)
Quartile 3	28,099	27,188	1.49 (1.46, 1.53)	1.30 (1.26, 1.33)
Quartile 4	28,894	26,525	1.46 (1.42, 1.50)	1.56 (1.52, 1.60)
Manganese				
Quartile 1	32,270	28,150	1.00 (reference)	1.00 (reference)
Quartile 2	32,420	22,723	0.78 (0.77, 0.80)	0.75 (0.73, 0.76)
Quartile 3	28,245	22,616	0.88 (0.86, 0.90)	0.78 (0.76, 0.80)
Quartile 4	29,539	26,618	1.02 (1.00, 1.05)	1.18 (1.15, 1.21)
Mercury				
Quartile 1	31,815	23,075	1.00 (reference)	1.00 (reference)
Quartile 2	29,247	25,376	1.20 (1.17, 1.23)	1.15 (1.12, 1.18)
Quartile 3	28,935	26,272	1.25 (1.22, 1.28)	1.13 (1.10, 1.16)
Quartile 4	32,477	25,384	1.08 (1.06, 1.11)	0.95 (0.92, 0.97)
Nickel				
Quartile 1	32,602	18,301	1.00 (reference)	1.00 (reference)
Quartile 2	28,815	25,789	1.54 (1.50, 1.58)	1.38 (1.34, 1.42)
Quartile 3	28,615	27,034	1.66 (1.62, 1.70)	1.47 (1.43, 1.51)
Quartile 4	32,442	28,983	1.54 (1.51, 1.58)	1.23 (1.20, 1.26)
Selenium				
Quartile 1	32,620	22,413	1.00 (reference)	1.00 (reference)
Quartile 2	31,727	25,451	1.11 (1.09, 1.14)	1.00 (0.97, 1.03)
Quartile 3	26,406	24,893	1.29 (1.26, 1.32)	1.03 (1.00, 1.06)
Quartile 4	31,721	27,350	1.20 (1.17, 1.23)	0.90 (0.88, 0.93)
PAHs				
Quartile 1	34,931	18,570	1.00 (reference)	1.00 (reference)
Quartile 2	29,218	24,831	1.60 (1.56, 1.64)	1.60 (1.56, 1.65)
Quartile 3	29,325	29,141	1.90 (1.85, 1.94)	1.66 (1.62, 1.71)
Quartile 4	29,000	27,565	1.76 (1.72, 1.81)	1.27 (1.23, 1.31)

^1^Adjusted for age, race, menopausal status, zip code level income and education, and parity status

The relationship between air toxics and breast density differed by menopausal status, with the associations tending to be stronger in women prior to menopause (Table 4). For some of the air toxics (cadmium, chromium, and selenium), there was a positive association with breast density in premenopausal women (e.g., cadmium Q4 vs Q1, OR = 1.26, 95% CI 1.20–1.32) although no positive association was evident in postmenopausal women. For some of the other air toxics (arsenic, cobalt, lead, manganese, nickel, and PAH), a positive association was apparent for both pre- and postmenopausal women but was more pronounced in premenopausal women (e.g., lead, premenopausal Q4 vs Q1, OR = 1.84, 95% CI 1.76–1.93; postmenopausal OR = 1.45, 95% CI 1.41–1.50).

**Table 4:** Air toxics and breast density by menopausal status at time of mammogram (Breast Cancer Surveillance Consortium, 2011)

Air toxics	Premenopausal			Postmenopausal			Interaction *p* value^2^
	Non-dense breasts	Dense breasts	Adjusted OR (95% CI)^1^	Non-dense breasts	Dense breasts	Adjusted OR (95% CI)^1^	
Arsenic							
Quartile 1	6645	11,106	1.00 (reference)	24,575	14,980	1.00 (reference)	< .0001
Quartile 2	7541	8645	0.77 (0.73, 0.80)	24,505	11,549	0.81 (0.78, 0.83)	
Quartile 3	5709	11,450	1.33 (1.27, 1.39)	21,859	15,314	1.22 (1.18, 1.26)	
Quartile 4	6272	11,578	1.37 (1.31, 1.44)	25,368	15,485	1.14 (1.11, 1.18)	
Cadmium							
Quartile 1	7212	8608	1.00 (reference)	26,631	15,671	1.00 (reference)	< .0001
Quartile 2	6482	10,043	1.09 (1.04, 1.14)	21,848	12,929	0.89 (0.86, 0.92)	
Quartile 3	5916	11,705	1.28 (1.22, 1.34)	21,721	13,969	0.90 (0.88, 0.93)	
Quartile 4	6557	12,423	1.26 (1.20, 1.32)	26,107	14,759	0.81 (0.78, 0.84)	
Chromium							
Quartile 1	7846	9445	1.00 (reference)	29,629	16,597	1.00 (reference)	< .0001
Quartile 2	6591	9191	1.22 (1.17, 1.28)	21,455	12,010	0.99 (0.96, 1.02)	
Quartile 3	5988	12,352	1.53 (1.46, 1.61)	22,703	15,717	1.10 (1.06, 1.13)	
Quartile 4	5742	11,791	1.33 (1.27, 1.40)	22,520	13,004	0.83 (0.81, 0.86)	
Cobalt							
Quartile 1	8427	7868	1.00 (reference)	28,128	12,269	1.00 (reference)	< .0001
Quartile 2	6149	10,203	1.33 (1.27, 1.40)	20,499	11,925	1.10 (1.06, 1.14)	
Quartile 3	5787	12,044	1.67 (1.59, 1.76)	22,628	15,712	1.34 (1.30, 1.39)	
Quartile 4	5804	12,664	1.98 (1.89, 2.08)	25,052	17,422	1.46 (1.41, 1.51)	
Lead							
Quartile 1	8163	9090	1.00 (reference)	27,414	13,285	1.00 (reference)	< .0001
Quartile 2	6231	10,217	1.13 (1.08, 1.19)	23,673	13,802	1.02 (0.99, 1.05)	
Quartile 3	5931	12,124	1.50 (1.43, 1.57)	22,168	15,064	1.22 (1.18, 1.26)	
Quartile 4	5842	11,348	1.84 (1.76, 1.93)	23,052	15,177	1.45 (1.41, 1.50)	
Manganese							
Quartile 1	6454	10,923	1.00 (reference)	25,816	17,227	1.00 (reference)	< .0001
Quartile 2	7463	9579	0.74 (0.71, 0.77)	24,957	13,144	0.76 (0.74, 0.78)	
Quartile 3	6208	10,509	0.91 (0.87, 0.96)	22,037	12,107	0.74 (0.71, 0.76)	
Quartile 4	6042	11,768	1.44 (1.37, 1.51)	23,497	14,850	1.09 (1.06, 1.12)	
Mercury							
Quartile 1	7233	10,002	1.00 (reference)	24,582	13,073	1.00 (reference)	0.01
Quartile 2	6131	10,818	1.17 (1.12, 1.22)	23,116	14,558	1.14 (1.10, 1.17)	
Quartile 3	6071	11,150	1.12 (1.07, 1.17)	22,864	15,122	1.14 (1.10, 1.17)	
Quartile 4	6732	10,809	0.97 (0.93, 1.01)	25,745	14,575	0.94 (0.91, 0.97)	
Nickel							
Quartile 1	7712	7222	1.00 (reference)	24,890	11,079	1.00 (reference)	< .0001
Quartile 2	6311	11,293	1.54 (1.47, 1.62)	22,504	14,496	1.30 (1.25, 1.34)	
Quartile 3	5821	11,305	1.66 (1.58, 1.75)	22,794	15,729	1.38 (1.33, 1.43)	
Quartile 4	6323	12,959	1.58 (1.50, 1.65)	26,119	16,024	1.10 (1.06, 1.14)	
Selenium							
Quartile 1	6605	7986	1.00 (reference)	26,015	14,427	1.00 (reference)	< .0001
Quartile 2	7458	10,826	1.08 (1.03, 1.13)	24,269	14,625	0.97 (0.95, 1.01)	
Quartile 3	5790	11,418	1.25 (1.19, 1.32)	20,616	13,475	0.95 (0.92, 0.98)	
Quartile 4	6314	12,549	1.21 (1.15, 1.28)	25,407	14,801	0.79 (0.77, 0.82)	
PAH							
Quartile 1	8361	7722	1.00 (reference)	26,570	10,848	1.00 (reference)	< .0001
Quartile 2	6258	10,801	1.79 (1.71, 1.88)	22,960	14,030	1.50 (1.45, 1.55)	
Quartile 3	5790	11,640	1.75 (1.67, 1.84)	23,535	17,501	1.61 (1.56, 1.66)	
Quartile 4	5758	12,616	1.54 (1.46, 1.62)	23,242	14,949	1.16 (1.12, 1.20)	

^1^Adjusted for age, race, zip code level income and education, and parity status

^2^*p* value from likelihood ratio test (df = 3) for models with and without inclusion of an interaction term between air toxic and menopausal status at time of mammogram

Associations were similar when we classified density using all four BI-RADS categories with associations most pronounced for exposure to arsenic, cobalt, and lead (Additional file 1: Table S2). There was evidence of effect measure modification by HT use for some of the metals, including lead and nickel (Additional file 1: Table S3). For example, the association with lead was apparent in women who were non-users of HT (OR = 1.60, 95% CI 1.56–1.66) but less pronounced in women who reported using HT (OR = 1.10, 95% 0.97–1.25). When considering potential effect measure modification by PAHs or selenium levels, we observed that some associations with density tended to be more pronounced in women who lived in areas below the median levels of PAHs or selenium levels (Additional file 1: Tables S4 and S5).

Further adjustment for rural/urban status and hormone replacement therapy did not substantially alter our results (data not shown). In analyses limited to women with non-missing BMI (which necessitated dropping registries that did not collect BMI), associations between air toxic levels and breast density were similar in models with and without adjustment for BMI, suggesting that even though BMI is related to breast density, it was not influential as a confounder in these analyses (Additional file 1: Table S6). Air toxic levels were not correlated with BMI (data not shown, all *r* < 0.15).

The WQS index was associated with breast density (OR = 1.22, 95% CI = 1.20, 1.24). A quartile increase in the WQS index resulted in a 20% higher odds of having dense breasts. Only two air toxics contributed meaningfully to the overall effect. These were lead (weight = 0.56) and cobalt (weight = 0.44).

## Discussion

In this large study population, we observed that living in areas of higher exposure to certain air toxics, especially lead and cobalt, was associated with higher odds of having dense breasts, particularly in premenopausal women. To the best of our knowledge, this is the first study to consider the relationship between air toxics and breast density. Breast density is a strong risk factor for breast cancer [2]; our findings suggest a role for environmental exposures in breast density and imply a possibly remediable role of these compounds in breast carcinogenesis.

Air pollution has been increasingly shown to be relevant for breast cancer [35]. Traffic-related pollution such as nitrogen dioxide (NO_2_) and PAH exposure has been suggestively related to breast cancer [19, 36–39]. Evidence is less consistent for hazardous air toxics [21, 40, 41] or particulate matter [36, 42, 43]. In the Sister Study cohort, living in areas of higher airborne cadmium, lead, mercury, and cobalt was related to a higher postmenopausal breast cancer risk [20]. However, few studies have considered the relationship between air pollution and breast density [12–14]. A previous study in the BCSC study population found that women with dense breasts had higher exposure to PM_2.5,_ which is a measure of a mixture of many compounds including toxic metals [14]_;_ these results are consistent with the findings presented here. None of these prior studies evaluated the relationship between breast density and metallic air toxics or PAH exposure. Both metals and PAHs are known to induce oxidative stress [30, 44, 45] and to cause endocrine disruption [22, 23, 46]. Both estrogenic activity [5] and oxidative stress [47] are hypothesized mechanisms by which these compounds may influence breast density and, thus, be relevant for breast cancer.

Air pollution is a complex mixture of many types of exposure, and a strength of this study is the inclusion of a mixture analytic approach to better mimic real life exposure to multiple correlated toxics. The use of the WQS method allows for the quantification of an overall effect—we observed 20% higher odds of dense breasts with each increasing quartile of the mixture exposure index. The WQS approach also permits the identification of the “bad actors” in the presence of correlated exposures. Lead and cobalt were identified to be the only weighted compounds of interest, suggesting they may drive the association, a finding that was consistent with the associations observed for those metals in the individual analysis. A limitation of WQS is that it only considers exposures that exhibit their effect in the same direction [33]; however, none of the air toxics exhibited a strong inverse association with density.

In epidemiologic studies, sources of PAH exposure, including outdoor [19, 39, 48] and indoor air pollution [49, 50], and adduct biomarkers of PAH exposure [51, 52], have been related to breast cancer risk. Although PAH exposure was related to breast density in our individual chemical analysis, in the WQS mixture analysis, it was downweighted to zero suggesting the observed association was actually driven by other correlated exposures.

Most of the research on heavy metals and breast cancer risk has focused on the role of cadmium [53]. The findings from these studies have been inconsistent; case-control studies with urinary cadmium measurements have reported consistently strong positive associations [53–57] whereas prospective cohort studies have not observed an association [58, 59]. Only one study evaluated the association between urinary lead and breast cancer risk; this case-control study found no evidence of an association [60]. Arsenic may be related to breast cancer risk in certain subgroups [61], but there is little evidence to date on the other toxic metals such as cobalt.

Inconsistent findings have been reported regarding the relation between urinary cadmium and breast density [10, 11] although a positive association was observed in one study that was limited to premenopausal women [11] consistent with the results reported here. The associations observed in this study tended to be stronger in women who were premenopausal. In our study population, 62% of premenopausal women had dense breasts whereas only 37% of postmenopausal women did. We also found that associations for some of the airborne metals tended to be higher in women who were non-HT users. This is consistent with a hypothesized estrogenic mechanism; women who were using HT may not be as susceptible to endocrine disrupting actions of the airborne toxics.

Airborne exposures are only a single exposure source, and participants may be exposed to these compounds through other sources including their diet, water sources, and tobacco smoke. We were unable to capture these other sources of exposure in this study. The BCSC did not collect information on cigarette smoking history, an important source of both PAHs and metals [18, 62]. Smoking appears to be associated with lower breast density, likely due to its anti-estrogenic effects [8, 9]. Therefore, a limitation of this study is that we could not evaluate whether this association varies based on cigarette smoking status. A limitation of WQS is that it does not identify interactions across exposures. Therefore, we evaluated some possible interactions of a priori interest including whether the associations with the remaining airborne metals and density varied by selenium exposure, as selenium has antioxidant properties [63] and may counteract the toxic effects of metals such as cadmium [29, 64]. The results for interactions with selenium were in line with that conjecture, with associations for many of the metals more pronounced in women who lived in areas below the median. In contrast, although we expected we might observe synergy between PAH exposure and toxic metals [30], the associations with the airborne toxics tended to be higher in those with low PAH exposure. This result could be due to differential residual confounding by other air pollutants, especially as the association between PAH and density observed here appeared to be driven by confounding with other air toxics as assessed by WQS.

We chose to use the most recently available NATA data release, rather than incorporating prior years of exposure because substantial changes have been made in the methodology over the years. The relevant etiologic window for exposures to alter breast density is unknown. This study was consequently cross-sectional by design, although it is likely that estimated 2011 air toxic levels may also represent past exposure. Previous studies have shown that density continues to change, even later in life [65], and that regimen changes in tamoxifen or HT use can alter breast density within 2–3 years [15, 16]. Thus, it is plausible that recent exposures are relevant for breast density. Another limitation of this study is the use of categorical BI-RADS categories rather than continuous measures of breast density. We did not have access to mammographic images and therefore could not use standardized and automated, objective measures of breast density. The BI-RADS categories have been previously shown to only have moderate interrater agreement [66]. For the main analyses, we grouped these categories into dense and non-dense outcomes for ease of interpretation and to ensure relevance for clinical practice. With the four-level categorization, the effects did tend to be stronger for BI-RADS 4 than for 3, so combining the two may obscure some of the effect. Future work in this area should consider the use of continuous, automated measures of breast density.

We adjusted for relevant covariates including race, zip code level education and income, which were important confounders in this study, and conducted sensitivity analyses for other potential confounding factors. Despite this, we cannot rule out the possibility that residual confounding may be present. Previous studies have reported that women in urban locations have elevated breast density compared to rural populations [67]. In sensitivity analyses, our results were consistent after the adjustment for urban/rural status, but further work is needed to better understand the potential relationships between urban/rural status, air pollution, and breast density. Similarly, BMI did not appear to be an important confounder although limiting to women with BMI data did change alter point estimates, likely due to study population differences in those with and without BMI data as BMI was only collected for some study sites.

This study was very well-powered, and the study population is diverse and generalizable. The air toxics were assessed by the EPA using a validated air pollution model. NATA estimates of lead have been previously related to body burden metal measures in children, supporting the validity of their air toxic models [68]. The dosimetry does have limitations, as exposure is estimated at the zip code level rather than individual level and there is likely some misclassification. Similarly, we also have no data on time spent outside, or at work in another zip code environment, use of air filtering devices in the home, window-opening behavior, etc., which factors would cause individual-level exposure variation. However imperfect, this modeled exposure data is the only resource to evaluate this question on a nationwide scale. Nevertheless, the actual effects of well-measured and well-timed exposures may be considerably stronger than what we report here.

## Conclusions

In this large, geographically diverse study, we found that women who live in areas of higher exposure to certain air toxics, especially lead and cobalt, were at higher odds of having dense breasts. These results were stronger in premenopausal women. This is the first study to evaluate the relationship between air toxic metals and PAHs in relation to breast density. Understanding the determinants of breast density is important, as women who have dense breasts are at a four to fivefold higher risk of developing breast cancer [2]. Breast cancer remains the most common cancer among women in the USA [3], and a better understanding of its environmental determinants could contribute both to preventative public health measures and to the elucidation of potential biologic mechanisms.

## Additional file

Additional file 1: **Figure S1.** Deciles of selected air toxics and breast density, Breast Cancer Surveillance Consortium, 2011. **Table S1.** Pearson correlation coefficients for air toxics, Breast Cancer Surveillance Consortium, 2011. **Table S2.** Air toxics and BI-RADS score, Breast Cancer Surveillance Consortium, 2011. **Table S3.** Air toxics and breast density by hormone therapy use (HT), Breast Cancer Surveillance Consortium, 2011. **Table S4.** Metallic air toxics and breast density by PAHs, Breast Cancer Surveillance Consortium, 2011. **Table S5.** Metallic air toxics and breast density by selenium, Breast Cancer Surveillance Consortium, 2011. **Table S6.** Air toxics and breast density when limited to participants with non-missing body mass index, Breast Cancer Surveillance Consortium, 2011. (DOCX 83 kb)

## Abbreviations

BCSC: Breast Cancer Surveillance Consortium
BI-RADS: Breast Imaging–Reporting and Data System
BMI: Body mass index
CI: 95% confidence intervals
EPA: Environmental Protection Agency
HIPAA: Health Insurance Portability and Accountability Act
HT: Hormone therapy
NATA: National Air Toxics Assessment
NO_2_: Nitrogen dioxide
OR: Odds ratio
PAHs: Polycyclic aromatic hydrocarbons
Q: Quartile
SCC: Statistical Coordinating Center
