# Supplementary material for: Airborne metals and polycyclic aromatic hydrocarbons in relation to mammographic breast density
Source: Breast Cancer Res. 2019 Feb 13;21:24. doi: 10.1186/s13058-019-1110-7 (PMC6373138; doi:10.1186/s13058-019-1110-7)
Supplement: Supplementary file 1 — Figure S1. Deciles of selected air toxics and breast density, Breast Cancer Surveillance Consortium, 2011. Table S1. Pearson correlation coefficients for air toxics, Breast Cancer Surveillance Consortium, 2011. Table S2. Air toxics and BI-RADS score, Breast Cancer Surveillance Consortium, 2011. Table S3. Air toxics and breast density by hormone therapy use (HT), Breast Cancer Surveillance Consortium, 2011. Table S4. Metallic air toxics and breast density by PAHs, Breast Cancer Surveillance Consortium, 2011. Table S5. Metallic air toxics and breast density by selenium, Breast Cancer Surveillance Consortium, 2011. Table S6. Air toxics and breast density when limited to participants with non-missing body mass index, Breast Cancer Surveillance Consortium, 2011. (DOCX 83 kb) [file 13058_2019_1110_MOESM1_ESM.docx]

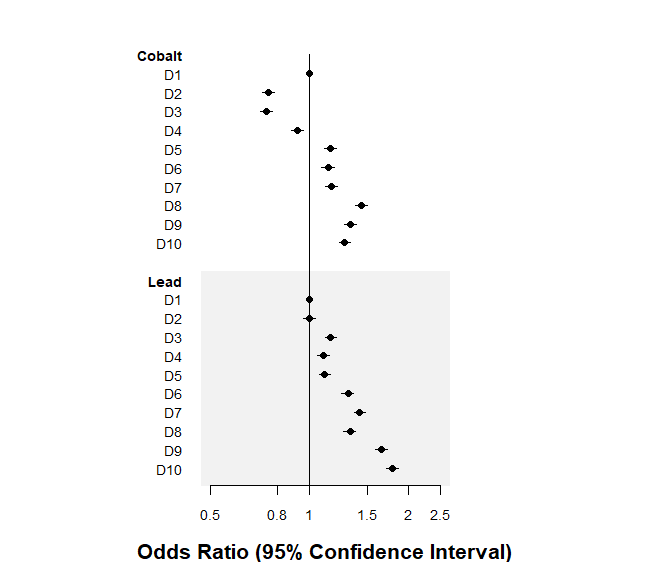


**Figure S1** Deciles of selected air toxics and breast density, Breast Cancer Surveillance Consortium, 2011.

**Table S1** Pearson correlation coefficients for air toxics, Breast Cancer Surveillance Consortium, 2011.

|  | Arsenic | Cadmium | Chromium | Cobalt | Lead | Manganese | Mercury | Nickel | Selenium | PAHs |
| --- | --- | --- | --- | --- | --- | --- | --- | --- | --- | --- |
| Arsenic | 1.00 |  |  |  |  |  |  |  |  |  |
| Cadmium | 0.36 | 1.00 |  |  |  |  |  |  |  |  |
| Chromium | 0.25 | 0.28 | 1.00 |  |  |  |  |  |  |  |
| Cobalt | 0.48 | 0.13 | 0.17 | 1.00 |  |  |  |  |  |  |
| Lead | 0.40 | 0.20 | 0.21 | 0.18 | 1.00 |  |  |  |  |  |
| Manganese | 0.44 | 0.30 | 0.19 | 0.15 | 0.47 | 1.00 |  |  |  |  |
| Mercury | 0.24 | 0.19 | 0.07 | -0.01 | -0.01 | 0.08 | 1.00 |  |  |  |
| Nickel | 0.69 | 0.21 | 0.27 | 0.31 | 0.19 | 0.18 | 0.17 | 1.00 |  |  |
| Selenium | 0.31 | 0.58 | 0.42 | 0.15 | 0.17 | 0.22 | 0.23 | 0.34 | 1.00 |  |
| PAHs | 0.29 | 0.44 | 0.44 | 0.17 | 0.05 | -0.01 | 0.24 | 0.41 | 0.86 | 1.00 |

**Table S2** Air toxics and BI-RADS score, Breast Cancer Surveillance Consortium, 2011.

|  |  | **BI-RADS 1** | **BI-RADS 2** | **BI-RADS 3** | **BI-RADS 4** | **Adjusted OR^1^  BI-RADS 2 vs. BI-RADS 1 (95% CI)** | **Adjusted OR^1^ BI-RADS 3 vs. BI-RADS 1 (95% CI)** | **Adjusted OR^1^ BI-RADS 4 vs. BI-RADS 1 (95% CI)** |
| --- | --- | --- | --- | --- | --- | --- | --- | --- |
|  |  |  |  |  |  |  |  |  |
| Arsenic |  |  |  |  |  |  |  |  |
|  | Quartile 1 | 7,108 | 24,112 | 21,180 | 4,906 | 1.00 (referent) | 1.00 (referent) | 1.00 (referent) |
|  | Quartile 2 | 7,223 | 24,823 | 16,911 | 3,283 | 1.00 (0.96, 1.04) | 0.80 (0.77, 0.83) | 0.71 (0.67, 0.76) |
|  | Quartile 3 | 6,498 | 21,070 | 21,188 | 5,576 | 0.95 (0.92, 0.99) | 1.16 (1.11, 1.21) | 1.42 (1.34, 1.50) |
|  | Quartile 4 | 7,788 | 23,852 | 21,183 | 5,880 | 0.92 (0.88, 0.95) | 1.05 (1.01, 1.10) | 1.52 (1.44, 1.61) |
| Cadmium |  |  |  |  |  |  |  |  |
|  | Quartile 1 | 7,797 | 26,046 | 20,289 | 3,990 | 1.00 (referent) | 1.00 (referent) | 1.00 (referent) |
|  | Quartile 2 | 6,678 | 21,652 | 18,247 | 4,725 | 0.92 (0.89, 0.96) | 0.87 (0.84, 0.91) | 0.97 (0.91, 1.02) |
|  | Quartile 3 | 5,744 | 21,893 | 20,428 | 5,246 | 1.02 (0.98, 1.06) | 1.01 (0.97, 1.06) | 1.06 (1.00, 1.12) |
|  | Quartile 4 | 8,398 | 24,266 | 21,498 | 5,684 | 0.79 (0.76, 0.82) | 0.76 (0.73, 0.79) | 0.81 (0.76, 0.86) |
| Chromium | |  |  |  |  |  |  |  |
|  | Quartile 1 | 10,842 | 26,633 | 21,121 | 4,921 | 1.00 (referent) | 1.00 (referent) | 1.00 (referent) |
|  | Quartile 2 | 5,433 | 22,613 | 17,695 | 3,506 | 1.61 (1.54, 1.67) | 1.54 (1.48, 1.61) | 1.35 (1.27, 1.43) |
|  | Quartile 3 | 5,518 | 23,173 | 22,384 | 5,685 | 1.58 (1.52, 1.64) | 1.73 (1.66, 1.80) | 1.67 (1.58, 1.77) |
|  | Quartile 4 | 6,824 | 21,438 | 19,262 | 5,533 | 1.21 (1.16, 1.26) | 1.11 (1.06, 1.16) | 1.08 (1.02, 1.14) |
| Cobalt |  |  |  |  |  |  |  |  |
|  | Quartile 1 | 8,480 | 28,075 | 17,607 | 2,530 | 1.00 (referent) | 1.00 (referent) | 1.00 (referent) |
|  | Quartile 2 | 5,835 | 20,813 | 18,050 | 4,078 | 1.00 (0.96, 1.04) | 1.16 (1.11, 1.21) | 1.35 (1.26, 1.45) |
|  | Quartile 3 | 5,973 | 22,442 | 22,028 | 5,728 | 1.03 (0.98, 1.07) | 1.41 (1.34, 1.47) | 1.96 (1.83, 2.09) |
|  | Quartile 4 | 8,329 | 22,527 | 22,777 | 7,309 | 0.82 (0.79, 0.85) | 1.25 (1.20, 1.30) | 2.32 (2.19, 2.47) |
| Lead |  |  |  |  |  |  |  |  |
|  | Quartile 1 | 8,719 | 26,858 | 18,951 | 3,424 | 1.00 (referent) | 1.00 (referent) | 1.00 (referent) |
|  | Quartile 2 | 7,037 | 22,867 | 19,098 | 4,921 | 1.04 (1.00, 1.08) | 1.06 (1.02, 1.1) | 1.22 (1.15, 1.29) |
|  | Quartile 3 | 6,814 | 21,285 | 21,216 | 5,972 | 1.02 (0.98, 1.06) | 1.26 (1.21, 1.32) | 1.63 (1.54, 1.72) |
|  | Quartile 4 | 6,047 | 22,847 | 21,197 | 5,328 | 1.19 (1.14, 1.23) | 1.67 (1.60, 1.74) | 2.51 (2.37, 2.66) |
| Manganese | |  |  |  |  |  |  |  |
|  | Quartile 1 | 7,041 | 25,229 | 22,785 | 5,365 | 1.00 (referent) | 1.00 (referent) | 1.00 (referent) |
|  | Quartile 2 | 8,036 | 24,384 | 18,431 | 4,292 | 0.81 (0.78, 0.84) | 0.64 (0.61, 0.66) | 0.6 (0.56, 0.63) |
|  | Quartile 3 | 7,679 | 20,566 | 17,533 | 5,083 | 0.73 (0.70, 0.76) | 0.61 (0.58, 0.63) | 0.65 (0.61, 0.68) |
|  | Quartile 4 | 5,861 | 23,678 | 21,713 | 4,905 | 1.07 (1.03, 1.12) | 1.22 (1.17, 1.27) | 1.4 (1.32, 1.48) |
| Mercury |  |  |  |  |  |  |  |  |
|  | Quartile 1 | 6,921 | 24,894 | 19,159 | 3,916 | 1.00 (referent) | 1.00 (referent) | 1.00 (referent) |
|  | Quartile 2 | 6,238 | 23,009 | 20,497 | 4,879 | 1.06 (1.02, 1.10) | 1.18 (1.14, 1.23) | 1.29 (1.22, 1.37) |
|  | Quartile 3 | 6,734 | 22,201 | 20,794 | 5,478 | 0.92 (0.89, 0.96) | 1.04 (1.00, 1.08) | 1.18 (1.12, 1.25) |
|  | Quartile 4 | 8,724 | 23,753 | 20,012 | 5,372 | 0.79 (0.76, 0.82) | 0.78 (0.75, 0.81) | 0.86 (0.81, 0.91) |
| Nickel |  |  |  |  |  |  |  |  |
|  | Quartile 1 | 7,179 | 25,423 | 16,104 | 2,197 | 1.00 (referent) | 1.00 (referent) | 1.00 (referent) |
|  | Quartile 2 | 5,956 | 22,859 | 20,964 | 4,825 | 0.98 (0.94, 1.03) | 1.30 (1.25, 1.36) | 1.85 (1.73, 1.97) |
|  | Quartile 3 | 6,187 | 22,428 | 21,673 | 5,361 | 0.95 (0.91, 0.99) | 1.34 (1.28, 1.40) | 2.03 (1.90, 2.17) |
|  | Quartile 4 | 9,295 | 23,147 | 21,721 | 7,262 | 0.66 (0.63, 0.68) | 0.81 (0.78, 0.85) | 1.50 (1.41, 1.60) |
| Selenium |  |  |  |  |  |  |  |  |
|  | Quartile 1 | 7,497 | 25,123 | 18,827 | 3,586 | 1.00 (referent) | 1.00 (referent) | 1.00 (referent) |
|  | Quartile 2 | 7,740 | 23,987 | 20,251 | 5,200 | 0.92 (0.89, 0.96) | 0.92 (0.88, 0.96) | 1.04 (0.98, 1.10) |
|  | Quartile 3 | 5,210 | 21,196 | 20,034 | 4,859 | 1.09 (1.04, 1.14) | 1.12 (1.07, 1.17) | 1.09 (1.02, 1.16) |
|  | Quartile 4 | 8,170 | 23,551 | 21,350 | 6,000 | 0.75 (0.72, 0.78) | 0.71 (0.68, 0.74) | 0.75 (0.71, 0.80) |
| PAH |  |  |  |  |  |  |  |  |
|  | Quartile 1 | 8,535 | 26,396 | 16,161 | 2,409 | 1.00 (referent) | 1.00 (referent) | 1.00 (referent) |
|  | Quartile 2 | 5,819 | 23,399 | 20,571 | 4,260 | 1.26 (1.21, 1.31) | 1.84 (1.76, 1.92) | 2.49 (2.34, 2.66) |
|  | Quartile 3 | 6,836 | 22,489 | 22,814 | 6,327 | 1.04 (1.00, 1.09) | 1.60 (1.54, 1.67) | 2.55 (2.40, 2.72) |
|  | Quartile 4 | 7,427 | 21,573 | 20,916 | 6,649 | 0.86 (0.82, 0.90) | 1.06 (1.01, 1.11) | 1.61 (1.50, 1.72) |

^1^Adjusted for age, race, menopausal status, zip-code level income and education, parity status

**Table S3** Air toxics and breast density by hormone therapy use (HT), Breast Cancer Surveillance Consortium, 2011.

|  |  | **No HT use** | | |  | **HT use** | | |  |
| --- | --- | --- | --- | --- | --- | --- | --- | --- | --- |
| **Air Toxics** | | **Non-dense breasts** | **Dense breasts** | **Adjusted OR (95%CI)^1^** |  | **Non-dense breasts** | **Dense breasts** | **Adjusted OR (95%CI)^1^** | **Interaction p-value^2^** |
|  |  |  |  |  |  |  |  |  |  |
| Arsenic |  |  |  |  |  |  |  |  |  |
|  | Quartile 1 | 26,802 | 22,664 | 1.00 (reference) |  | 1,928 | 2,053 | 1.00 (reference) |  |
|  | Quartile 2 | 28,168 | 17,819 | 0.79 (0.77, 0.81) |  | 1,946 | 1,394 | 0.75 (0.68, 0.83) |  |
|  | Quartile 3 | 23,916 | 23,598 | 1.25 (1.21, 1.28) |  | 1,093 | 1,261 | 1.15 (1.04, 1.28) |  |
|  | Quartile 4 | 27,741 | 24,147 | 1.21 (1.18, 1.25) |  | 906 | 906 | 1.02 (0.91, 1.15) | 0.06 |
| Cadmium | |  |  |  |  |  |  |  |  |
|  | Quartile 1 | 29,651 | 20,989 | 1.00 (reference) |  | 2,159 | 1,950 | 1.00 (reference) |  |
|  | Quartile 2 | 24,757 | 20,159 | 0.95 (0.93, 0.98) |  | 1,517 | 1,532 | 0.93 (0.84, 1.03) |  |
|  | Quartile 3 | 23,899 | 22,971 | 1.04 (1.01, 1.07) |  | 942 | 881 | 0.75 (0.66, 0.84) |  |
|  | Quartile 4 | 28,320 | 24,109 | 0.95 (0.92, 0.97) |  | 1,255 | 1,251 | 0.79 (0.70, 0.88) | <.0001 |
| Chromium | |  |  |  |  |  |  |  |  |
|  | Quartile 1 | 32,861 | 22,725 | 1.00 (reference) |  | 1,844 | 1,748 | 1.00 (reference) |  |
|  | Quartile 2 | 24,713 | 18,552 | 1.05 (1.02, 1.08) |  | 1,705 | 1,575 | 0.93 (0.84, 1.03) |  |
|  | Quartile 3 | 24,943 | 25,013 | 1.24 (1.21, 1.28) |  | 1,122 | 1,134 | 0.84 (0.75, 0.94) |  |
|  | Quartile 4 | 24,110 | 21,938 | 0.99 (0.96, 1.02) |  | 1,202 | 1,157 | 0.76 (0.68, 0.85) | <.0001 |
| Cobalt |  |  |  |  |  |  |  |  |  |
|  | Quartile 1 | 32,832 | 18,013 | 1.00 (reference) |  | 1,849 | 1,234 | 1.00 (reference) |  |
|  | Quartile 2 | 22,531 | 19,106 | 1.18 (1.14, 1.21) |  | 1,658 | 1,669 | 1.16 (1.03, 1.30) |  |
|  | Quartile 3 | 24,538 | 24,582 | 1.45 (1.40, 1.49) |  | 1,149 | 1,237 | 1.31 (1.16, 1.48) |  |
|  | Quartile 4 | 26,726 | 26,527 | 1.61 (1.57, 1.66) |  | 1,217 | 1,474 | 1.45 (1.29, 1.64) | 0.1 |
| Lead |  |  |  |  |  |  |  |  |  |
|  | Quartile 1 | 31,774 | 19,823 | 1.00 (reference) |  | 1,872 | 1,590 | 1.00 (reference) |  |
|  | Quartile 2 | 25,680 | 20,940 | 1.06 (1.03, 1.09) |  | 1,887 | 1,787 | 0.98 (0.89, 1.08) |  |
|  | Quartile 3 | 24,114 | 23,752 | 1.31 (1.27, 1.34) |  | 1,390 | 1,517 | 1.12 (1.01, 1.25) |  |
|  | Quartile 4 | 25,059 | 23,713 | 1.60 (1.56, 1.65) |  | 724 | 720 | 1.10 (0.97, 1.25) | <.0001 |
| Manganese | |  |  |  |  |  |  |  |  |
|  | Quartile 1 | 27,653 | 24,193 | 1.00 (reference) |  | 2,064 | 2,360 | 1.00 (reference) |  |
|  | Quartile 2 | 28,175 | 19,904 | 0.75 (0.73, 0.77) |  | 2,027 | 1,632 | 0.76 (0.69, 0.83) |  |
|  | Quartile 3 | 24,806 | 20,098 | 0.79 (0.77, 0.81) |  | 1,267 | 1,154 | 0.75 (0.67, 0.83) |  |
|  | Quartile 4 | 25,993 | 24,033 | 1.21 (1.17, 1.24) |  | 515 | 468 | 0.86 (0.74, 0.99) | <.0001 |
| Mercury | |  |  |  |  |  |  |  |  |
|  | Quartile 1 | 28,102 | 20,569 | 1.00 (reference) |  | 1,492 | 1,176 | 1.00 (reference) |  |
|  | Quartile 2 | 25,464 | 22,386 | 1.15 (1.12, 1.18) |  | 1,232 | 1,266 | 1.22 (1.09, 1.37) |  |
|  | Quartile 3 | 24,915 | 22,959 | 1.12 (1.09, 1.15) |  | 1,384 | 1,562 | 1.31 (1.17, 1.46) |  |
|  | Quartile 4 | 28,146 | 22,314 | 0.94 (0.92, 0.97) |  | 1,765 | 1,610 | 1.05 (0.95, 1.17) | 0.005 |
| Nickel |  |  |  |  |  |  |  |  |  |
|  | Quartile 1 | 29,272 | 16,292 | 1.00 (reference) |  | 1,848 | 1,299 | 1.00 (reference) |  |
|  | Quartile 2 | 24,620 | 22,532 | 1.39 (1.35, 1.43) |  | 1,414 | 1,511 | 1.25 (1.11, 1.41) |  |
|  | Quartile 3 | 24,678 | 23,846 | 1.49 (1.44, 1.53) |  | 1,038 | 1,121 | 1.23 (1.09, 1.40) |  |
|  | Quartile 4 | 28,057 | 25,558 | 1.23 (1.20, 1.27) |  | 1,573 | 1,683 | 1.20 (1.07, 1.34) | 0.06 |
| Selenium | |  |  |  |  |  |  |  |  |
|  | Quartile 1 | 28,556 | 19,520 | 1.00 (reference) |  | 1,939 | 1,605 | 1.00 (reference) |  |
|  | Quartile 2 | 28,166 | 22,601 | 1.00 (0.98, 1.03) |  | 1,406 | 1,310 | 0.96 (0.86, 1.07) |  |
|  | Quartile 3 | 22,535 | 21,838 | 1.05 (1.02, 1.08) |  | 1,212 | 1,388 | 0.93 (0.82, 1.05) |  |
|  | Quartile 4 | 27,370 | 24,269 | 0.91 (0.89, 0.94) |  | 1,316 | 1,311 | 0.82 (0.73, 0.92) | 0.4 |
| PAH |  |  |  |  |  |  |  |  |  |
|  | Quartile 1 | 31,468 | 16,788 | 1.00 (reference) |  | 1,667 | 986 | 1.00 (reference) |  |
|  | Quartile 2 | 25,619 | 22,213 | 1.60 (1.56, 1.65) |  | 799 | 730 | 1.42 (1.24, 1.62) |  |
|  | Quartile 3 | 25,015 | 25,255 | 1.65 (1.60, 1.69) |  | 1,618 | 1,962 | 1.78 (1.58, 2.01) |  |
|  | Quartile 4 | 24,525 | 23,972 | 1.25 (1.21, 1.29) |  | 1,789 | 1,936 | 1.44 (1.27, 1.63) | 0.4 |

^1^Adjusted for age, race, menopausal status, zip-code level income and education, parity status

^2^ P-value from likelihood ratio test for models with and without inclusion of an interaction term between air toxic and hormone therapy use

**Table S4** Metallic air toxics and breast density by PAHs, Breast Cancer Surveillance Consortium, 2011.

|  |  | **< median PAHs** | | |  | **≥ median PAHs** | | |  |  |
| --- | --- | --- | --- | --- | --- | --- | --- | --- | --- | --- |
| **Metals** | | **Non-dense breasts** | **Dense breasts** | **Adjusted OR (95%CI)^1^** |  | **Non-dense breasts** | **Dense breasts** | **Adjusted OR (95%CI)^1^** | **Interaction p-value^2^** | |
|  |  |  |  |  |  |  |  |  |  |  |
| Arsenic |  |  |  |  |  |  |  |  |  |  |
|  | Quartile 1 | 18,088 | 11,580 | 1.00 (reference) |  | 13,132 | 14,506 | 1.00 (reference) |  |  |
|  | Quartile 2 | 20,472 | 9,287 | 0.71 (0.69, 0.74) |  | 11,574 | 10,907 | 0.90 (0.87, 0.94) |  |  |
|  | Quartile 3 | 12,423 | 11,880 | 1.29 (1.24, 1.34) |  | 15,145 | 14,884 | 1.14 (1.09, 1.18) |  |  |
|  | Quartile 4 | 13,166 | 10,654 | 1.20 (1.15, 1.24) |  | 18,474 | 16,409 | 1.14 (1.10, 1.19) | <.0001 |  |
| Cadmium | |  |  |  |  |  |  |  |  |  |
|  | Quartile 1 | 25,012 | 14,617 | 1.00 (reference) |  | 8,831 | 9,662 | 1.00 (reference) |  |  |
|  | Quartile 2 | 17,478 | 11,691 | 0.98 (0.95, 1.01) |  | 10,852 | 11,281 | 0.78 (0.74, 0.81) |  |  |
|  | Quartile 3 | 11,402 | 9,906 | 1.15 (1.11, 1.20) |  | 16,235 | 15,768 | 0.73 (0.70, 0.76) |  |  |
|  | Quartile 4 | 10,257 | 7,187 | 1.15 (1.11, 1.20) |  | 22,407 | 19,995 | 0.63 (0.60, 0.65) | <.0001 |  |
| Chromium | |  |  |  |  |  |  |  |  |  |
|  | Quartile 1 | 28,097 | 15,663 | 1.00 (reference) |  | 9,378 | 10,379 | 1.00 (reference) |  |  |
|  | Quartile 2 | 19,753 | 12,285 | 1.08 (1.04, 1.11) |  | 8,293 | 8,916 | 0.91 (0.87, 0.95) |  |  |
|  | Quartile 3 | 13,795 | 13,185 | 1.60 (1.54, 1.66) |  | 14,896 | 14,884 | 0.71 (0.69, 0.74) |  |  |
|  | Quartile 4 | 2,504 | 2,268 | 1.51 (1.41, 1.62) |  | 25,758 | 22,527 | 0.63 (0.60, 0.65) | <.0001 |  |
| Cobalt |  |  |  |  |  |  |  |  |  |  |
|  | Quartile 1 | 33,533 | 18,065 | 1.00 (reference) |  | 3,022 | 2,072 | 1.00 (reference) |  |  |
|  | Quartile 2 | 13,864 | 9,093 | 1.09 (1.05, 1.13) |  | 12,784 | 13,035 | 0.92 (0.85, 0.98) |  |  |
|  | Quartile 3 | 9,688 | 9,696 | 1.65 (1.59, 1.72) |  | 18,727 | 18,060 | 0.93 (0.87, 1.00) |  |  |
|  | Quartile 4 | 7,064 | 6,547 | 1.71 (1.64, 1.79) |  | 23,792 | 23,539 | 1.05 (0.98, 1.12) | <.0001 |  |
| Lead |  |  |  |  |  |  |  |  |  |  |
|  | Quartile 1 | 31,111 | 17,021 | 1.00 (reference) |  | 4,466 | 5,354 | 1.00 (reference) |  |  |
|  | Quartile 2 | 11,011 | 5,173 | 0.86 (0.83, 0.90) |  | 18,893 | 18,846 | 0.88 (0.84, 0.93) |  |  |
|  | Quartile 3 | 8,365 | 8,840 | 1.61 (1.55, 1.68) |  | 19,734 | 18,348 | 0.87 (0.83, 0.92) |  |  |
|  | Quartile 4 | 13,662 | 12,367 | 1.77 (1.70, 1.84) |  | 15,232 | 14,158 | 1.04 (0.99, 1.09) | <.0001 |  |
| Manganese | |  |  |  |  |  |  |  |  |  |
|  | Quartile 1 | 18,176 | 12,587 | 1.00 (reference) |  | 14,094 | 15,563 | 1.00 (reference) |  |  |
|  | Quartile 2 | 16,398 | 6,923 | 0.59 (0.57, 0.61) |  | 16,022 | 15,800 | 0.86 (0.83, 0.89) |  |  |
|  | Quartile 3 | 11,948 | 8,066 | 0.81 (0.78, 0.84) |  | 16,297 | 14,550 | 0.77 (0.75, 0.80) |  |  |
|  | Quartile 4 | 17,627 | 15,825 | 1.17 (1.13, 1.22) |  | 11,912 | 10,793 | 1.05 (1.01, 1.10) | <.0001 |  |
| Mercury | |  |  |  |  |  |  |  |  |  |
|  | Quartile 1 | 24,340 | 15,192 | 1.00 (reference) |  | 7,475 | 7,883 | 1.00 (reference) |  |  |
|  | Quartile 2 | 19,072 | 14,799 | 1.18 (1.14, 1.21) |  | 10,175 | 10,577 | 1.05 (1.01, 1.10) |  |  |
|  | Quartile 3 | 11,283 | 8,697 | 1.14 (1.10, 1.19) |  | 17,652 | 17,575 | 0.98 (0.94, 1.02) |  |  |
|  | Quartile 4 | 9,454 | 4,713 | 0.83 (0.79, 0.86) |  | 23,023 | 20,671 | 0.87 (0.83, 0.90) | <.0001 |  |
| Nickel |  |  |  |  |  |  |  |  |  |  |
|  | Quartile 1 | 31,580 | 17,566 | 1.00 (reference) |  | 1,022 | 735 | 1.00 (reference) |  |  |
|  | Quartile 2 | 16,857 | 12,787 | 1.19 (1.15, 1.23) |  | 11,958 | 13,002 | 1.14 (1.03, 1.27) |  |  |
|  | Quartile 3 | 12,838 | 11,056 | 1.35 (1.30, 1.40) |  | 15,777 | 15,978 | 1.12 (1.01, 1.25) |  |  |
|  | Quartile 4 | 2,874 | 1,992 | 1.11 (1.04, 1.18) |  | 29,568 | 26,991 | 0.92 (0.83, 1.02) | <.0001 |  |
| Selenium | |  |  |  |  |  |  |  |  |  |
|  | Quartile 1 | 23,805 | 14,458 | 1.00 (reference) |  | 8,815 | 7,955 | 1.00 (reference) |  |  |
|  | Quartile 2 | 22,533 | 15,582 | 0.93 (0.90, 0.96) |  | 9,194 | 9,869 | 1.14 (1.09, 1.19) |  |  |
|  | Quartile 3 | 10,023 | 7,784 | 1.01 (0.97, 1.05) |  | 16,383 | 17,109 | 0.91 (0.87, 0.95) |  |  |
|  | Quartile 4 | 7,788 | 5,577 | 1.18 (1.13, 1.24) |  | 23,933 | 21,773 | 0.72 (0.70, 0.76) | <.0001 |  |

^1^Adjusted for age, race, menopausal status, zip-code level income and education, parity status

^2^ P-value from likelihood ratio test for models with and without inclusion of an interaction term between remaining air toxics and median PAH levels

**Table S5** Metallic air toxics and breast density by selenium, Breast Cancer Surveillance Consortium, 2011.

|  |  | **< median selenium** | | |  | **≥ median selenium** | | |  |  |
| --- | --- | --- | --- | --- | --- | --- | --- | --- | --- | --- |
| **Metals** | | **Non-dense breasts** | **Dense breasts** | **Adjusted OR (95%CI)^1^** |  | **Non-dense breasts** | **Dense breasts** | **Adjusted OR (95%CI)^1^** | **Interaction p-value^2^** | |
|  |  |  |  |  |  |  |  |  |  |  |
| Arsenic |  |  |  |  |  |  |  |  |  |  |
|  | Quartile 1 | 18,620 | 12,243 | 1.00 (reference) |  | 12,600 | 13,843 | 1.00 (reference) |  |  |
|  | Quartile 2 | 21,923 | 11,336 | 0.83 (0.80, 0.86) |  | 10,123 | 8,858 | 0.76 (0.73, 0.79) |  |  |
|  | Quartile 3 | 14,552 | 15,877 | 1.46 (1.40, 1.51) |  | 13,016 | 10,887 | 0.88 (0.85, 0.92) |  |  |
|  | Quartile 4 | 9,252 | 8,408 | 1.28 (1.23, 1.34) |  | 22,388 | 18,655 | 1.00 (0.96, 1.04) | <.0001 |  |
| Cadmium | |  |  |  |  |  |  |  |  |  |
|  | Quartile 1 | 33,576 | 23,977 | 1.00 (reference) |  | 267 | 302 | 1.00 (reference) |  |  |
|  | Quartile 2 | 22,459 | 16,045 | 0.88 (0.85, 0.9) |  | 5,871 | 6,927 | 0.91 (0.77, 1.09) |  |  |
|  | Quartile 3 | 6,870 | 6,888 | 1.11 (1.06, 1.15) |  | 20,767 | 18,786 | 0.77 (0.64, 0.91) |  |  |
|  | Quartile 4 | 1,442 | 954 | 1.07 (0.98, 1.17) |  | 31,222 | 26,228 | 0.68 (0.57, 0.81) | <.0001 |  |
| Chromium | |  |  |  |  |  |  |  |  |  |
|  | Quartile 1 | 35,832 | 25,376 | 1.00 (reference) |  | 1,643 | 666 | 1.00 (reference) |  |  |
|  | Quartile 2 | 19,019 | 13,221 | 1.08 (1.05, 1.11) |  | 9,027 | 7,980 | 2.09 (1.89, 2.31) |  |  |
|  | Quartile 3 | 7,127 | 6,684 | 1.15 (1.10, 1.20) |  | 21,564 | 21,385 | 2.37 (2.15, 2.61) |  |  |
|  | Quartile 4 | 2,369 | 2,583 | 1.36 (1.28, 1.45) |  | 25,893 | 22,212 | 1.76 (1.59, 1.94) | <.0001 |  |
| Cobalt |  |  |  |  |  |  |  |  |  |  |
|  | Quartile 1 | 36,499 | 20,077 | 1.00 (reference) |  | 56 | 60 | 1.00 (reference) |  |  |
|  | Quartile 2 | 9,663 | 8,147 | 1.33 (1.28, 1.38) |  | 16,985 | 13,981 | 0.74 (0.50, 1.08) |  |  |
|  | Quartile 3 | 8,198 | 8,685 | 1.55 (1.48, 1.61) |  | 20,217 | 19,071 | 0.90 (0.61, 1.32) |  |  |
|  | Quartile 4 | 9,987 | 10,955 | 1.73 (1.67, 1.80) |  | 20,869 | 19,131 | 0.96 (0.65, 1.42) | 0.009 |  |
| Lead |  |  |  |  |  |  |  |  |  |  |
|  | Quartile 1 | 30,694 | 17,184 | 1.00 (reference) |  | 4,883 | 5,191 | 1.00 (reference) |  |  |
|  | Quartile 2 | 12,797 | 8,228 | 1.13 (1.09, 1.17) |  | 17,107 | 15,791 | 0.84 (0.80, 0.89) |  |  |
|  | Quartile 3 | 13,613 | 15,301 | 1.66 (1.60, 1.71) |  | 14,486 | 11,887 | 0.80 (0.76, 0.84) |  |  |
|  | Quartile 4 | 7,243 | 7,151 | 1.57 (1.51, 1.64) |  | 21,651 | 19,374 | 1.21 (1.15, 1.28) | <.0001 |  |
| Manganese | |  |  |  |  |  |  |  |  |  |
|  | Quartile 1 | 24,645 | 18,926 | 1.00 (reference) |  | 7,625 | 9,224 | 1.00 (reference) |  |  |
|  | Quartile 2 | 20,363 | 12,012 | 0.80 (0.78, 0.83) |  | 12,057 | 10,711 | 0.71 (0.68, 0.74) |  |  |
|  | Quartile 3 | 12,491 | 10,020 | 0.92 (0.89, 0.96) |  | 15,754 | 12,596 | 0.65 (0.62, 0.68) |  |  |
|  | Quartile 4 | 6,848 | 6,906 | 1.19 (1.14, 1.24) |  | 22,691 | 19,712 | 1.07 (1.02, 1.12) | <.0001 |  |
| Mercury | |  |  |  |  |  |  |  |  |  |
|  | Quartile 1 | 20,970 | 13,302 | 1.00 (reference) |  | 10,845 | 9,773 | 1.00 (reference) |  |  |
|  | Quartile 2 | 17,212 | 13,282 | 1.15 (1.11, 1.19) |  | 12,035 | 12,094 | 1.07 (1.03, 1.12) |  |  |
|  | Quartile 3 | 14,179 | 12,340 | 1.24 (1.19, 1.28) |  | 14,756 | 13,932 | 0.93 (0.90, 0.97) |  |  |
|  | Quartile 4 | 11,986 | 8,940 | 1.22 (1.17, 1.26) |  | 20,491 | 16,444 | 0.75 (0.72, 0.78) | <.0001 |  |
| Nickel |  |  |  |  |  |  |  |  |  |  |
|  | Quartile 1 | 32,170 | 18,054 | 1.00 (reference) |  | 432 | 247 | 1.00 (reference) |  |  |
|  | Quartile 2 | 16,103 | 13,698 | 1.23 (1.19, 1.27) |  | 12,712 | 12,091 | 1.75 (1.48, 2.07) |  |  |
|  | Quartile 3 | 9,445 | 10,105 | 1.56 (1.5, 1.61) |  | 19,170 | 16,929 | 1.69 (1.43, 2.00) |  |  |
|  | Quartile 4 | 6,629 | 6,007 | 1.40 (1.34, 1.46) |  | 25,813 | 22,976 | 1.45 (1.23, 1.72) | <.0001 |  |
| Selenium | |  |  |  |  |  |  |  |  |  |
|  | Quartile 1 | 33,611 | 18,123 | 1.00 (reference) |  | 1,320 | 447 | 1.00 (reference) |  |  |
|  | Quartile 2 | 12,727 | 11,917 | 1.48 (1.43, 1.53) |  | 16,491 | 12,914 | 2.57 (2.29, 2.88) |  |  |
|  | Quartile 3 | 15,634 | 15,225 | 1.66 (1.61, 1.72) |  | 13,691 | 13,916 | 2.66 (2.36, 2.98) |  |  |
|  | Quartile 4 | 2,375 | 2,599 | 1.80 (1.69, 1.92) |  | 26,625 | 24,966 | 2.09 (1.86, 2.35) | <.0001 |  |

^1^Adjusted for age, race, menopausal status, zip-code level income and education, parity status

^2^ P-value from likelihood ratio test for models with and without inclusion of an interaction term between remaining air toxics and median PAH levels

**Table S6** Air toxics and breast density when limited to participants with non-missing body mass index, Breast Cancer Surveillance Consortium, 2011.

|  |  | **In all study participants** | | |  | **In participants with non-missing BMI** | | | | |  |  |
| --- | --- | --- | --- | --- | --- | --- | --- | --- | --- | --- | --- | --- |
| **Metals** |  | **Non-dense breasts** | **Dense breasts** | **Adjusted OR (95% CI)^1^** |  | **Non-dense breasts** | **Dense breasts** | **Adjusted OR (95% CI)^1^** |  | **Adjusted OR^1^ + BMI (95%CI)** |  |  |
|  |  |  |  |  |  |  |  |  |  |  |  |  |
| Arsenic |  |  |  |  |  |  |  |  |  |  |  |  |
|  | Quartile 1 | 31,220 | 26,086 | 1.00 (reference) |  | 26,268 | 22,880 | 1.00 (reference) |  | 1.00 (reference) |  |  |
|  | Quartile 2 | 32,046 | 20,194 | 0.79 (0.77, 0.81) |  | 19,698 | 14,326 | 0.81 (0.78, 0.83) |  | 0.82 (0.80, 0.85) |  |  |
|  | Quartile 3 | 27,568 | 26,764 | 1.25 (1.21, 1.28) |  | 16,711 | 15,883 | 1.15 (1.12, 1.19) |  | 1.27 (1.23, 1.31) |  |  |
|  | Quartile 4 | 31,640 | 27,063 | 1.20 (1.17, 1.23) |  | 14,130 | 11,352 | 0.96 (0.93, 1.00) |  | 0.97 (0.93, 1.00) |  |  |
| Cadmium | |  |  |  |  |  |  |  |  |  |  |  |
|  | Quartile 1 | 33,843 | 24,279 | 1.00 (reference) |  | 24,830 | 20,151 | 1.00 (reference) |  | 1.00 (reference) |  |  |
|  | Quartile 2 | 28,330 | 22,972 | 0.94 (0.92, 0.97) |  | 17,926 | 14,564 | 0.8 (0.78, 0.82) |  | 0.71 (0.69, 0.74) |  |  |
|  | Quartile 3 | 27,637 | 25,674 | 1.00 (0.98, 1.03) |  | 12,613 | 11,348 | 0.73 (0.70, 0.76) |  | 0.62 (0.60, 0.65) |  |  |
|  | Quartile 4 | 32,664 | 27,182 | 0.92 (0.90, 0.95) |  | 21,438 | 18,378 | 0.71 (0.69, 0.73) |  | 0.57 (0.56, 0.59) |  |  |
| Chromium | |  |  |  |  |  |  |  |  |  |  |  |
|  | Quartile 1 | 37,475 | 26,042 | 1.00 (reference) |  | 35,853 | 25,123 | 1.00 (reference) |  | 1.00 (reference) |  |  |
|  | Quartile 2 | 28,046 | 21,201 | 1.05 (1.02, 1.08) |  | 12,671 | 10,949 | 1.05 (1.01, 1.08) |  | 0.94 (0.91, 0.98) |  |  |
|  | Quartile 3 | 28,691 | 28,069 | 1.21 (1.18, 1.24) |  | 12,875 | 13,665 | 1.07 (1.03, 1.10) |  | 0.95 (0.91, 0.98) |  |  |
|  | Quartile 4 | 28,262 | 24,795 | 0.96 (0.93, 0.99) |  | 15,408 | 14,704 | 0.88 (0.85, 0.91) |  | 0.74 (0.71, 0.76) |  |  |
| Cobalt |  |  |  |  |  |  |  |  |  |  |  |  |
|  | Quartile 1 | 36,555 | 20,137 | 1.00 (reference) |  | 24,511 | 14,450 | 1.00 (reference) |  | 1.00 (reference) |  |  |
|  | Quartile 2 | 26,648 | 22,128 | 1.18 (1.14, 1.21) |  | 17,472 | 14,622 | 1.07 (1.03, 1.11) |  | 1.06 (1.02, 1.10) |  |  |
|  | Quartile 3 | 28,415 | 27,756 | 1.44 (1.40, 1.48) |  | 16,991 | 17,154 | 1.33 (1.28, 1.38) |  | 1.38 (1.33, 1.43) |  |  |
|  | Quartile 4 | 30,856 | 30,086 | 1.6 (1.56, 1.64) |  | 17,833 | 18,215 | 1.43 (1.39, 1.48) |  | 1.48 (1.43, 1.54) |  |  |
| Lead |  |  |  |  |  |  |  |  |  |  |  |  |
|  | Quartile 1 | 35,577 | 22,375 | 1.00 (reference) |  | 26,512 | 17,899 | 1.00 (reference) |  | 1.00 (reference) |  |  |
|  | Quartile 2 | 29,904 | 24,019 | 1.05 (1.03, 1.08) |  | 22,775 | 20,171 | 1.06 (1.03, 1.10) |  | 1.07 (1.04, 1.11) |  |  |
|  | Quartile 3 | 28,099 | 27,188 | 1.3 (1.26, 1.33) |  | 16,947 | 16,511 | 1.25 (1.21, 1.29) |  | 1.32 (1.28, 1.37) |  |  |
|  | Quartile 4 | 28,894 | 26,525 | 1.56 (1.52, 1.60) |  | 10,573 | 9,860 | 1.4 (1.35, 1.46) |  | 1.53 (1.47, 1.59) |  |  |
| Manganese | |  |  |  |  |  |  |  |  |  |  |  |
|  | Quartile 1 | 32,270 | 28,150 | 1.00 (reference) |  | 28,500 | 25,749 | 1.00 (reference) |  | 1.00 (reference) |  |  |
|  | Quartile 2 | 32,420 | 22,723 | 0.75 (0.73, 0.76) |  | 21,231 | 17,283 | 0.77 (0.75, 0.79) |  | 0.75 (0.73, 0.77) |  |  |
|  | Quartile 3 | 28,245 | 22,616 | 0.78 (0.76, 0.80) |  | 18,613 | 14,754 | 0.68 (0.66, 0.70) |  | 0.61 (0.59, 0.63) |  |  |
|  | Quartile 4 | 29,539 | 26,618 | 1.18 (1.15, 1.21) |  | 8,463 | 6,655 | 0.9 (0.87, 0.94) |  | 0.86 (0.82, 0.90) |  |  |
| Mercury | |  |  |  |  |  |  |  |  |  |  |  |
|  | Quartile 1 | 31,815 | 23,075 | 1.00 (reference) |  | 17,398 | 12,862 | 1.00 (reference) |  | 1.00 (reference) |  |  |
|  | Quartile 2 | 29,247 | 25,376 | 1.15 (1.12, 1.18) |  | 15,826 | 13,371 | 1.14 (1.10, 1.18) |  | 1.18 (1.13, 1.22) |  |  |
|  | Quartile 3 | 28,935 | 26,272 | 1.13 (1.10, 1.16) |  | 18,775 | 17,617 | 1.21 (1.17, 1.25) |  | 1.28 (1.24, 1.32) |  |  |
|  | Quartile 4 | 32,477 | 25,384 | 0.95 (0.92, 0.97) |  | 24,808 | 20,591 | 1.01 (0.98, 1.04) |  | 1.02 (0.98, 1.05) |  |  |
| Nickel |  |  |  |  |  |  |  |  |  |  |  |  |
|  | Quartile 1 | 32,602 | 18,301 | 1.00 (reference) |  | 19,459 | 12,244 | 1.00 (reference) |  | 1.00 (reference) |  |  |
|  | Quartile 2 | 28,815 | 25,789 | 1.38 (1.34, 1.42) |  | 18,427 | 16,110 | 1.15 (1.11, 1.19) |  | 1.19 (1.15, 1.23) |  |  |
|  | Quartile 3 | 28,615 | 27,034 | 1.47 (1.43, 1.51) |  | 14,659 | 13,547 | 1.18 (1.14, 1.23) |  | 1.25 (1.20, 1.30) |  |  |
|  | Quartile 4 | 32,442 | 28,983 | 1.23 (1.20, 1.26) |  | 24,262 | 22,540 | 1.05 (1.02, 1.09) |  | 1.01 (0.97, 1.04) |  |  |
| Selenium | |  |  |  |  |  |  |  |  |  |  |  |
|  | Quartile 1 | 32,620 | 22,413 | 1.00 (reference) |  | 25,301 | 18,543 | 1.00 (reference) |  | 1.00 (reference) |  |  |
|  | Quartile 2 | 31,727 | 25,451 | 1.00 (0.97, 1.03) |  | 17,198 | 13,149 | 0.86 (0.83, 0.89) |  | 0.77 (0.74, 0.80) |  |  |
|  | Quartile 3 | 26,406 | 24,893 | 1.03 (1.00, 1.06) |  | 11,755 | 12,242 | 0.84 (0.81, 0.87) |  | 0.68 (0.65, 0.71) |  |  |
|  | Quartile 4 | 31,721 | 27,350 | 0.90 (0.88, 0.93) |  | 22,553 | 20,507 | 0.75 (0.72, 0.77) |  | 0.58 (0.56, 0.60) |  |  |
| PAH |  |  |  |  |  |  |  |  |  |  |  |  |
|  | Quartile 1 | 34,931 | 18,570 | 1.00 (reference) |  | 22,728 | 11,765 | 1.00 (reference) |  | 1.00 (reference) |  |  |
|  | Quartile 2 | 29,218 | 24,831 | 1.60 (1.56, 1.65) |  | 11,971 | 8,483 | 1.41 (1.36, 1.47) |  | 1.49 (1.43, 1.55) |  |  |
|  | Quartile 3 | 29,325 | 29,141 | 1.66 (1.62, 1.71) |  | 19,477 | 20,630 | 1.82 (1.76, 1.88) |  | 2.02 (1.95, 2.09) |  |  |
|  | Quartile 4 | 29,000 | 27,565 | 1.27 (1.23, 1.31) |  | 22,631 | 23,563 | 1.46 (1.41, 1.51) |  | 1.39 (1.34, 1.45) |  |  |
| ^1^Adjusted for age, race, menopausal status, zip-code level income and education, parity status | | | | | | | | | | |  |  |
